# From light-harvesting to photoprotection: structural basis of the dynamic switch of the major antenna complex of plants (LHCII)

**DOI:** 10.1038/srep15661

**Published:** 2015-10-23

**Authors:** Nicoletta Liguori, Xavier Periole, Siewert J. Marrink, Roberta Croce

**Affiliations:** 1 Department of Physics and Astronomy and Institute for Lasers, Life and Biophotonics, Faculty of Sciences, De Boelelaan 1081, 1081 HV, Amsterdam, The Netherlands; 2 Groningen Biomolecular Sciences and Biotechnology Institute & Zernike Institute for Advanced Materials, University of Groningen, Nijenborgh 7, 9747 AG Groningen, The Netherlands

## Abstract

Light-Harvesting Complex II (LHCII) is largely responsible for light absorption and excitation energy transfer in plants in light-limiting conditions, while in high-light it participates in photoprotection. It is generally believed that LHCII can change its function by switching between different conformations. However, the underlying molecular picture has not been elucidated yet. The available crystal structures represent the quenched form of the complex, while solubilized LHCII has the properties of the unquenched state. To determine the structural changes involved in the switch and to identify potential quenching sites, we have explored the structural dynamics of LHCII, by performing a series of microsecond Molecular Dynamics simulations. We show that LHCII in the membrane differs substantially from the crystal and has the signatures that were experimentally associated with the light-harvesting state. Local conformational changes at the N-terminus and at the xanthophyll neoxanthin are found to strongly correlate with changes in the interactions energies of two putative quenching sites. In particular conformational disorder is observed at the terminal emitter resulting in large variations of the excitonic coupling strength of this chlorophyll pair. Our results strongly support the hypothesis that light-harvesting regulation in LHCII is coupled with structural changes.

Higher plants evolved a natural capacity to modulate photosynthetic activity in response to varying light and other environmental conditions^1^. In low light they need to harvest every available photon to sustain life, while in high light they dissipate the energy absorbed in excess to avoid photodamage. Light-Harvesting Complexes (LHCs) are pigment-protein systems responsible for photon absorption and transfer of the excitation energy to the reaction center, where charge separation occurs^2^.

All LHCs share a highly homologous protein sequence^3^ and a very similar folding^4^,^5^,^6^. A typical LHC architecture is exemplified by the major LHC complex, LHCII, for which two high-resolution structures are available^4^,^6^. One LHCII monomer binds a total of 18 pigments: 6 chlorophylls *b* (Chlb), 8 chlorophylls *a* (Chla) and 4 xanthophyll (carotenoids, here abbreviated as Cars, containing oxygen atoms) molecules: luteins (Lut 1 and Lut 2), violaxanthin (Vio) and neoxanthin (Neo)^6^. For the nomenclature of LHCII protein domains, Chls and Cars we refer to Liu *et al.*^6^. The pigments are embedded in the protein matrix and are mainly coordinated by the three transmembrane helixes that represent the common motif of the LHC structure^2^,^5^,^6^. In the following LHCII in the membrane is sometime indicated as “solubilized” LHCII to distinguish it from the “crystal” LHCII. A simplified scheme of LHCII can be found in Fig. 1A–D.

The network of chlorophyll (Chl) and carotenoid (Car) interactions in LHCs, described as excitonic interactions^7^, is naturally designed not only to increase the absorption cross section of the system, but also to ensure fast excitation energy transfer while maintaining a relatively long Chl singlet excited state lifetime to deliver the energy quanta to the reaction center with high efficiency^2^. In addition to their role in light harvesting, in high-light conditions LHCs are involved in photoprotection, lowering the level of excited states in the membrane through a process known as Non-Photochemical Quenching (NPQ)^8^. In the photoprotective state, their chlorophyll excited state lifetime is significantly shortened and thus the probability of forming singlet oxygen species is highly reduced, preventing photo-oxidative damage in the plant. It is generally believed that the double functionality of the LHCs is the result of different conformations of the complexes that can “switch” from the light-harvesting to the photoprotective state^9^,^10^.

Interestingly, fluorescence experiments have shown that crystallized LHCII has the properties of the photoprotective/quenched state^11^,^12^,^13^. The structure reported for LHCII crystals is then hypothesized to be different than the one(s) of solubilized LHCII, which instead is characterized by a high fluorescence yield (light-harvesting/unquenched state)^11^,^13^. It should be mentioned that the quenched conformation observed in the crystal is not due to interactions between different complexes, as the complexes in the crystal are functionally separated^11^. Raman spectroscopy has indicated that LHCII in the crystals, in aggregates or gels in the absence of detergent, all examples of strongly quenched species, assumes a similar conformation^11^,^14^,^15^. These studies have systematically reported a series of structural differences respect to the solubilized form. More in detail crystallized LHCII shows distortion at the xanthophyll neoxanthin and the presence of a strong hydrogen bond at a Chlb-formyl site in the crystal, tentatively assigned to either Chlb606 or b607, in contrast to the solubilized/unquenched LHCII. Similar changes have been observed *in vivo* in NPQ conditions^14^. Based on these findings, a correlation has been proposed between these structural changes and the induction of dissipative states^8^,^14^.

Conformational changes are supposed to control the energetics in LHCs by varying pigment-pigment interactions and thus opening quenching channels^8^,^9^,^10^,^16^. Strengthening of excitonic interactions between Chl-Chl and/or Chl-Car sites has been widely proposed as the origin of quenching, and various pigment clusters have been suggested as quenching sites^11^,^14^,^17^,^18^,^19^. However, the absence of the structure of the solubilized complex has limited the possibility to validate these proposals.

Classic Molecular Dynamics simulations (MDs) on various photosynthetic systems were shown to be a powerful tool to study functional aspects of these complexes in native- or experimental-mimicking environments such as model membranes^20^,^21^,^22^ or micelles^23^. Amongst these studies, recent simulations applied to a cyanobacterial Photosystem II^20^ and a bacterial reaction center^22^ embedded in model membranes, have shown that MD is able to reproduce the different conformations of pigments and protein required to predict realistic pigments site energies^20^ and activation barriers of electron transfer processes^22^.

In this work we have performed a series of microsecond Molecular Dynamics simulations (MDs) to follow the dynamics of a monomer of LHCII from higher plants^6^ in a native-mimicking membrane with the aim of monitoring changes in protein structure, xanthophyll conformation, and pigment-pigment interactions that can be related to the switch from the crystal/quenched state to the solubilized/light-harvesting state of the complex. Although LHCII trimer is the most abundant form of this complex in the thylakoid, LHCII monomers have been proposed to be present in native conditions^24^,^25^. Also, LHCII shares high sequence homology with LHC minor antennae, which are all present in monomeric form in the membrane and they all share the same ability of LHCII to switch between different fluorescence states^26^. The exploration of the microsecond time-range via MDs allowed us to access events, such as conformational changes, otherwise invisible in the nanosecond timescale. We report the molecular picture of a series of systematic structural changes corroborating several previous experimental findings, and suggesting that our structures are relaxing in the timescale here explored towards the light-harvesting state. Moreover, analyses of the structural changes observed in our MDs combined with excitonic calculations show that LHCII in the membrane is a dynamic system, whose conformational changes correlate with, and might control, transitions into the different energy states of the complex.

## Results and Discussion

### Crystal and solubilized LHCII show different structural features

Our results show that the pigment-protein complex in the membrane reproduces the overall structural flexibility observed in the crystal, as captured by its B-factor (Fig. 2A,B and Methods in SI). The high rigidity at the alpha-helix core-domains, and the high flexibility of the solvent exposed regions (Fig. 2B) that we observe for the solubilized LHCII are in agreement with EPR measurements^27^. Indeed a rigid core has been proposed to be crucial for maintaining the proper architecture of Chl-Chl and Chl-Car interactions for efficient light harvesting^27^,^28^.

To compare more in detail the structure of LHCII in the membrane and in the crystal, the Root Mean Square Deviation (RMSD) evolution has been calculated for the different structural domains of the protein (Supplementary Figure S3). It can be shown that while during the simulation the helix regions maintain an almost identical structure to that of the crystal, the stromal loop and especially the N-terminus of the protein strongly deviate from the crystal structure. EPR/ESR studies on LHCII and on the homologous antenna CP29 have previously shown that the N-terminus is indeed highly flexible^27^,^29^, adopting various conformations in solution, suggesting that this domain is constitutively highly disordered. It can be concluded that compared to the crystal, LHCII in the membrane maintains the rigid alpha-helix core structure, while differs in the peripheral region, especially in the stromal-exposed domains.

### LHCII carotenoids: mobile outside, steady inside

**Figure 1:**
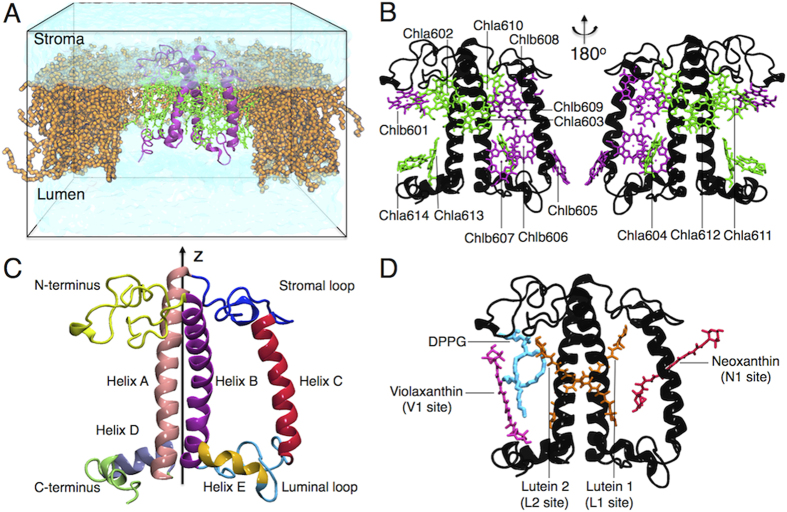
(**A**–**D**). Molecular architecture of LHCII. **A**) Scheme of the simulation box containing water (transparent cyan), the POPC membrane (orange) and the full pigment-binding LHCII system (apoprotein in black, cofactors in green). Lipids surrounding LHCII complex have been removed for clarity. (**B**) Two different side-views of the LHCII-complex showing the chlorophylls network. Eight Chla (green), six Chlb (purple) and the apoprotein (black) are shown. Chlorophyll phytol tails are not shown. (**C**) LHCII apoprotein structural domains. The different regions are rendered in different colors. The pseudo C2-symmetry axis of the protein (z-axis of the protein) is shown^6^. (**D**) Side view of LHCII showing the positions of the four carotenoids (binding sites are indicated in parentheses) and the lipid (DPPG).

The four xanthophylls associated with LHCII have different roles (Cars binding sites are reported in Figs 1D and 3). They are essential for the stability of the complex (Lut)^30^, take part in light harvesting (Lut and Neo)^31^,^32^,^33^, and participate in photoprotection. This last function is fulfilled directly by quenching Chl triplets (Lut 1 and 2)^34^ or Chl singlets (Lut)^14^,^17^,^18^,^35^, or indirectly by providing a readily available substrate for the Violaxanthin-De-Epoxidase (VDE), which converts Vio to Zeaxanthin, a factor necessary for the full NPQ development^36^. Changes in the carotenoid arrangement can thus have a large effect on the functionality of the complex. The experimental data show on both crystal and solubilized LHCII that Lut 1, Lut 2 and Neo are stably associated with the complex^4^,^6^,^37^, although Raman data show that the organization of Neo differs between crystallized and solubilized LHCII^11^. The violaxanthin in the V1 site (See Fig. 1D) was shown to be loosely bound to the complex as the occupancy of V1 in trimeric LHCII depends on the solubilization protocol^33^,^37^, and this site is empty upon monomerization^33^,^37^.

Our simulations are in complete agreement with the experimental results on solubilized LHCII. By measuring the angle between their S_2_ ← S_0_ transition dipole moment, taken parallel to the central portion of the polyene chain^6^,^38^, and the z-axis of the protein we find that Lut 1 and Lut 2 are not only stably associated with the complex but their motion is strongly limited (Fig. 3, Supplementary Table S3). In contrast, we systematically observe large deviations from their position in the crystal for Neo and Vio. Our simulations show that, in agreement with experimental results^39^, the portion of Neo buried inside LHCII is stably anchored to TYR112 (Supplementary Figure S5 and Table S2), while the portion that protrudes outside the protein undergoes distortion and bending (Fig. 3, Supplementary Table S3 and Video S2). Notably the portion of Neo exposed to the environment is kinked to different extents in the three available crystal structures of LHCs^4^,^5^,^6^. Also, based on the large changes in the Raman band at 953 cm^−1^, Neo has been predicted to adopt a different configuration in the light-harvesting/solubilized form respect to the crystal/quenched form^11^,^14^,^15^.

Our results indicate that the angle between the Neo dipole moment and the z-axis of the protein, which is ~60 degrees in the crystal structure, equilibrates to an average of ~90 degrees in the simulations, meaning that the molecule is highly kinked (Fig. 3, Supplementary Table S3 and Video S2). At variance with the other carotenoids, but again in agreement with experimental results^33^,^37^, Vio appears to be only loosely bound to LHCII. We observed from partial to complete detachment of Vio in all the simulations (Fig. 3 and Supplementary Video S2). In particular, Vio moves out of its binding site in the crystal, where it lies parallel to the protein axis, and re-orients almost perpendicularly to the protein axis (Fig. 3 and Supplementary Table S3). It should be noted that the orientation of Vio perpendicular to the protein axis and parallel to the membrane plane, as portrayed by our simulations, was also observed experimentally^40^.

### Energy disorder in LHCII

The large absorption cross section and efficient energy transfer cascade inside LHCs has been naturally engineered via a specific geometry of Chl-Chl and Chl-Car interactions^2^. Delocalization of photo-excitations over different pigments inside one or multiple LHCs is reached via strong dipole-dipole interactions, also called excitonic interactions, between the chromophores^7^,^41^,^42^. The magnitude of such interactions depends on the distance cube (power of −3), and on the relative orientations of their transition dipole moments^7^. Thermal motions of the single pigments are expected to induce fluctuations around the average interaction energy and eventual displacement of the pigments with respect to their position in the crystal may strongly influence the average coupling strength.

Conformational changes of the protein, as those expected to take place during the switch between light-harvesting and quenched states, are thus predicted to play an important role in modifying the spectroscopic properties of the pigments^9^,^16^,^26^. However, no information about the effect of the protein dynamics on these interactions is available, leading some researchers to challenge the possibility that different conformations of LHCs are responsible for the different functionalities^12^.

To determine to which extent the dynamics of the complex can alter pigment-pigment interactions, we have thus calculated the time-dependent excitonic coupling strengths between the strongest interacting pigments and compared them with the values obtained from the crystal structure^6^,^41^ (see Methods in SI). Here we have used a point-dipole approximation to compute all the coupling values, as done by Liu *et al.* for the LHCII crystal^6^. Although especially in the case of short intermolecular distances other methods might be more accurate^43^,^44^, the large differences respect to the crystal value that we observe for some of the Chl pairs are an indication of the large re-organization freedom at these chlorophyll sites.

### Chl-Chl clusters

We found systematic deviations from the crystal values for most of the chlorophyll pairs in all the simulations (Supplementary Table S4) with the exception of Chlb608-Chla610 (Fig. 4) and Chla613-Chla614 (Supplementary Figure S6 and Table S4). The largest deviations were observed for Chlb607-Chlb606 and Chla611-Chla612, which showed an increase and decrease, respectively, of the interaction energies by ~60% and ~50% on average compared to the crystal value (Fig. 4, Supplementary Table S4). Notably these two clusters have been proposed as putative quenching sites in the complex^11^,^14^,^42^. In the following we discuss in detail the changes observed at these sites.

### Chlb607-Chlb606 cluster

In Raman experiments on LHCII-crystals, a narrow band at 1639 cm^−1^, otherwise missing in unquenched/solubilized samples, was assigned to the presence of a hydrogen bond at a Chlb-formyl site, tentatively attributed to the Chlb607-Chlb606 cluster^11^,^15^. This local conformational change was then suggested to possibly lead to a strong exciton dimer, functional in energy quenching^11^,^15^. In agreement with the Raman results, upon solubilization in the membrane we observe a systematic and reproducible loss of an H-bond present in the crystal structure at the Chlb607-Chlb606 site (Supplementary Figure S10 and Table S6). An example of this event is shown in Supplementary Video S3, where it can be observed that the H-bond between the Chlb607-formyl group and GLN131 breaks, and the GLN131 switches to coordinate Chlb606. This is also in agreement with mutational analysis studies that have indicated that GLN131 is the ligand of Chlb606 in solution^45^. However, we do not find correlation between the loss of the H-bond and the variation in coupling strength between the two Chls (Supplementary Table S7). Also, the significant increase in this coupling strength in the membrane compared to the situation in the crystal (Fig. 4 and Supplementary Table S4), suggests that the observed conformational change of this particular Chl cluster is unlikely to be responsible for the functional switch.

### Chla611-Chla612 cluster

Chla611 and Chla612 form the strongest exciton cluster in the crystal and are responsible for the lowest energy form of LHCII^42^,^44^,^45^. In the functional Photosystem II supercomplexes this Chl cluster is responsible for the transfer of the excitation energy from LHCII to CP29 and to the core^46^, and it is thus an optimal site for light-harvesting regulation. Indeed, this Chl pair alone or in combination with Lut 1 was proposed as site of quenching^14^,^47^. Our simulations show that this cluster is highly dynamic. Our findings reconcile with the observation that Chla611-Chla612 are in slightly different conformations in the two crystal structures^4^,^6^,^44^, and with experimental evidences that show the influence of thermal motions on the Chla611-Chla612 interaction in solubilized LHCII^44^,^48^. More importantly, our simulations reveal that the interaction energy between these two Chls decreases in the membrane as compared to the crystal, making this site an excellent candidate for the quenching site. Consequently, the factors influencing this cluster might be important in the regulation of light-harvesting in LHCII. In Simulation A after ~300 ns we observed a strong deviation of the interaction energy between these two Chls from the crystal value (Fig. 4), which is due to the re-orientation and movement of Chla612 towards Chla611 (Supplementary Figure S8 and Supplementary Video S4). As anticipated, the extent of the variations in the excitonic coupling in Simulation A are likely to be overestimated by the point-dipole method used here due to the short dipole-dipole distances between the chlorophylls reached in this simulation. Application of a more accurate method such as the extended-dipole^43^,^44^ indeed results in reduced fluctuations (see Supplementary Figure S9). However a strong reduction in interaction energy is again found over time, similarly to what is observed by using the point-dipole method (Fig. 4). Video S4 shows that DPPG coordinates via its phosphate the central Mg of Chla611, is itself coordinated to LYS182 (Helix B) and TYR44 (N-terminus)^6^, and interacts with other residues at the N-terminus of the protein^4^,^6^, suggesting that changes in the N-terminus can influence this pigment cluster. By plotting the RMSD of each simulation per single residue (Supplementary Figure S4), it is possible to infer that the N-terminus relaxes to different conformations, which all differ from that of the crystal. We can thus test the effect of the organization of the N-terminus on this Chl cluster. Interestingly, the N-terminus movement changes the network of interactions around the ligand of Chla611^4^,^6^ (Supplementary Video S4 shows an example of it). The analyses show that different coupling states of the Chla611-Chla612 cluster correlate to different conformations of the N-terminus (0.99 Pearson correlation, see Fig. 5 and Supplementary Table S7). In particular, the shorter the distance between the N-terminus and the Chla611-ligand (DPPG), the weaker the interaction energy between Chla611 and Chla612 is. Considering the key role of Chla611-Chla612 in the energy cascade of LHCII^42^,^44^, structural disorder at this site might be at the origin of the molecular switching of LHCII^9^. Moreover, it is known that the N-terminus is involved in protein-protein interactions^49^ and it can be expected that external factors such as changes in the connectivity between complexes, which occur as a result of membrane reorganization in stress conditions^50^,^51^, or phosphorylation of the N-terminus as observed during state transitions^52^, can trigger and stabilize the conformational switch.

### Chl-Car clusters

Due to their very short-living excited state, carotenoids are in principle ideal quenchers^41^. Indeed several authors have reported that energy dissipation can occur via interactions between Lut 1 or Lut 2 and neighboring Chls. This interaction is expected to involve the S1 forbidden state of the carotenoids^14^,^18^,^41^, which cannot be unambiguously calculated yet. We thus proceed by calculating the interaction between the Chls and the carotenoid transition dipole moment S_2 _← S_0_ as done in previous studies^6^,^38^. Although these values cannot be directly related to the quenching, they give information about the possibility for rearrangement of the different Chl and Car clusters, which is an essential requirement for switching between light-harvesting and quenched states^8^,^9^,^10^.

**Figure 2:**
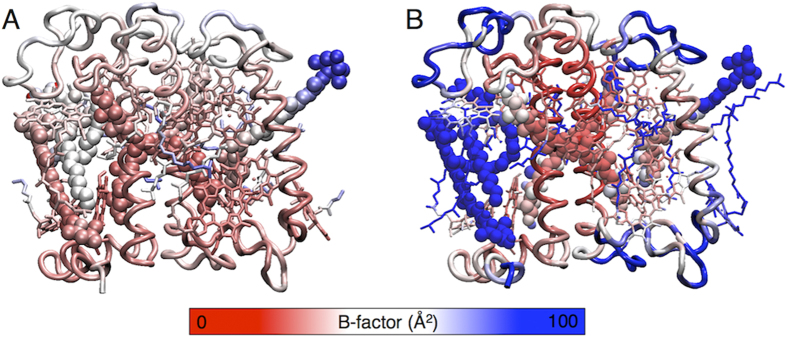
(**A,B**). LHCII flexibility. LHCII structure as from the crystal^6^ (**A**) and as from simulation A (**B**), colored by their B-factor (Debye-Waller or temperature factor, see Methods in SI). B-factor values are shown with colors ranging from red (low fluctuations) to blue (high fluctuations). The single LHCII components are shown as tubes (protein), sticks (Chls) and Van der Waals spheres (Cars and DPPG). Note that in the crystal, the coordinates for a set of Chl phytol tails^6^ are missing (see Methods in SI).

In contrast to what we found for the Chl-Chl pairs, we observed that most Chl-Car interactions, and therefore their relative distance and orientation, are conserved when compared to the crystal interactions (Supplementary Figure S7 and Table S5). Interestingly, we observe significant modulations only for Chla603-Lut 2 and especially for Chla612-Lut 1 (Fig. 4, Supplementary Figure S7 and Table S5). It is worth noticing that Chla603, which is located at the interface between monomers, might be less flexible in LHCII trimers (Fig. 2). Together with the dynamics reported at the Chla611-Chla612 site (see above), our results suggest that the Chla611-Chla612-Lut 1 cluster possesses all the characteristics for being a site of light-harvesting regulation in LHCII.

### Neoxanthin can act as reporter of a quenched conformation of LHCII

A correlation between Neo distortions and a quenched conformation has been observed experimentally^14^,^53^. We have thus tested possible correlations between the ensemble of different orientations of Neo and the Chl-Chl and Chl-Car coupling states computed. We find that such correlation exists only in case of Chla603-Lut 2 (Fig. 5 and Supplementary Table S7), which notably has been pinpointed as one of the putative quenching sites^17^,^54^,^55^. The data indicate that the structures showing the highest bending of Neo also show the highest deviations from the coupling strength at this site in the crystal.

**Figure 3:**
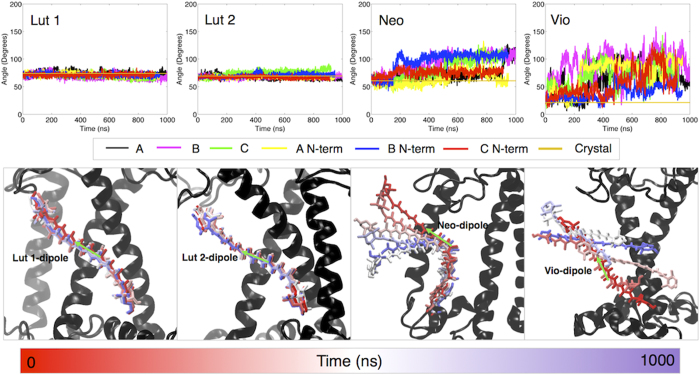
Carotenoid dynamics. Top panel: time-evolution of the angle between the dipole moment of each carotenoid (as indicated in panel) present in LHCII and the protein z-axis (as defined in Fig. 1C) in each simulation. Different colors indicate different simulations as shown in the legend below the top panel. The dipole moment is directed as shown by the green arrow in the bottom panel. The time evolution has been computed over the full trajectories of each simulation. The value of the angle calculated from the crystal is also reported (gold). Bottom panel: time-dependent conformations extracted from Simulation A. Six different conformations for each carotenoid have been extracted at regular intervals from the trajectory of simulation A (see legend at the bottom for the color code representing the time) and are here overlaid to the initial conformation of the apoprotein (in black).

**Figure 4:**
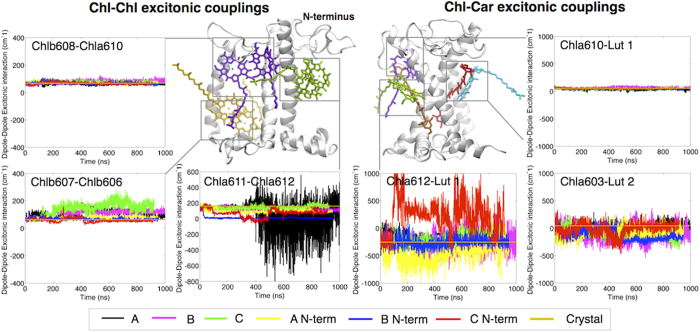
Pigment-pigment interaction dynamics. Time-evolution of the interaction energies for selected examples of excitonically strongly coupled pigments. We report three examples of Chl-Chl excitonic couplings (left side of the panel) and three examples of Chl-Car excitonic coupling (right side of the panel). The time-dependent couplings have been computed over the whole trajectories of the full set of simulations, as reported in the legend (bottom side of the panel for the color code). In each plot the coupling value calculated from the crystal is also reported. In the central part of the panel a representation of the structure of LHCII with indicated the Chl-Chl and Chl-Car pairs corresponding to the plots. The apoprotein is represented in white, chlorophylls are represented in either purple, green, orange or cyan, and carotenoids (Lut 1 and Lut 2) are represented respectively in orange or red.

Based on the fact that the neoxanthin protrudes out of LHCII into the membrane, the strong correlation observed here can be caused by the environment influencing both Neo and the Chla603-Lut 2 cluster organization. Another explanation for the strong correlation can rely on the direct effect of Neo in activating the re-arrangement of these pigments through its bending motion, therefore acting as a trigger for the conformational change. Indeed a direct cause-effect relationship between changes in the neoxanthin structure, and the switch to the quenched conformation, has been previously suggested^8^,^14^,^53^. We therefore tested this hypothesis by investigating whether the gradual tilting of Neo is synchronized with the variations of the Chla603-Lut 2 coupling strength. We did that by plotting per each simulation the Chla603-Lut 2 interaction energies as a function of the different angles of Neo form the protein axis, both computed over the full trajectory (as reported respectively in Fig. 3 and 4). No correlation between the two events is observed in the analysis (Supplementary Figure S11 and Table S8), suggesting that Neo distortions cannot directly induce changes in the exciton manifold. It is likely that Neo, due to its flexible structure and its exposure to the outer environment, is prone to be affected by the same environmental changes that are believed to induce dissipative states and then in the experiments acts as a reporter, but not as a trigger, for the conformational change involving the putative quenching site Chla603-Lut 2.

## Conclusions

**Figure 5:**
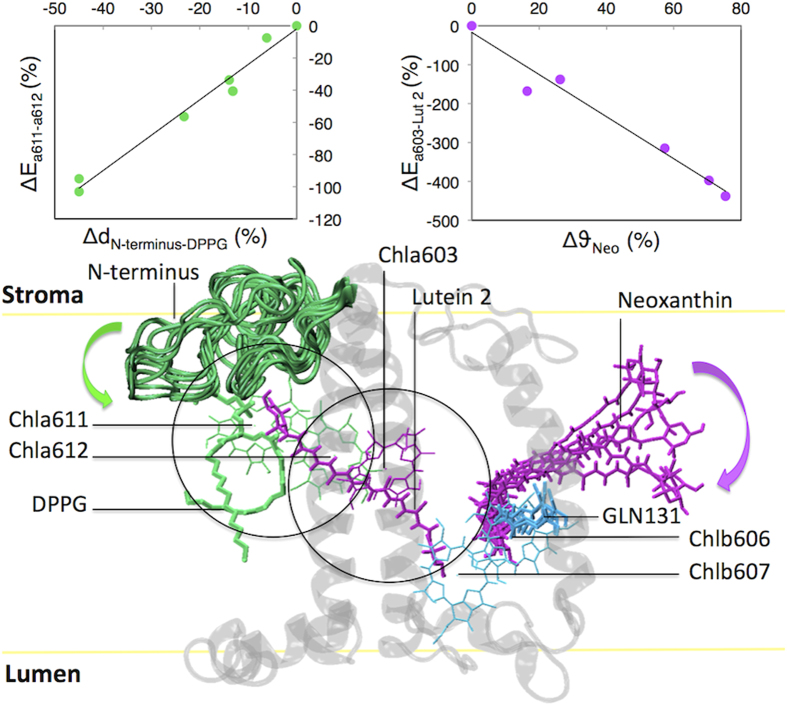
Model of the conformational switch of LHCII. Upper panel: on the left the variation of excitonic coupling at the Chla611-Chla612 cluster (∆E_a611-a612_) is plotted in function of the decrease in distance between the N-terminus and DPPG (∆d_N-terminus-DPPG_), which is the ligand of Chla611. On the right the variation of excitonic coupling at the Chla603-Lut 2 cluster (∆E_a603-Lut 2_) is plotted in function of the extent of bending of the xanthophyll neoxanthin (∆ϑ_Neo_). The (0,0) point represents the crystal. Trend lines show the best fit (0.99 Pearson coefficient for the plot on the left, −0.98 Pearson coefficient for the plot on the right). Note that two points on the second graph overlap (See Table S3 and S5). Lower panel: summary of the conformational changes observed in our simulations going from the crystal to the membrane-solubilized form of LHCII. Different colors represent the different domains of LHCII involved in the switch: in green the N-terminus conformational changes associated with the Chla612-Chla612-DPPG cluster; in magenta the neoxanthin bending (indicated by a red arrow) associated with the Chla603-Lut 2 cluster; in cyan the GLN131 conformational change associated with the Chlb607-Chlb606 domain (H-bond loss between the Chlb607-formyl and GLN131 oxygen). Protein is shown in transparent black, the membrane is schematically represented in yellow. Chlorophylls phytol tails are not shown for clarity. Snapshots extracted at regular intervals from simulation A, depict various examples of the conformations of N-terminus, neoxanthin and GLN131. Black circles highlight the regions in which a strong correlation has been observed.

By investigating *in silico* the dynamics of LHCII in the microsecond time range, we observed systematic changes from the crystal structure matching experimental observations, which indicate that the structure of LHCII in the model membrane has the structural characteristics expected for the light-harvesting state of the complex. A model of the solubilized LHCII conformation, summarizing the conformational changes observed in this work is presented in Fig. 5. LHCII in the membrane shows a highly disordered N-terminus and loss of an H-bond at the Chlb607-Chlb606 site. Neoxanthin is also strongly kinked compared to the crystal structure.

More important, our simulations uncovered a strong correlation between the structural disorder at the N-terminus and the energetic disorder of the lowest energy site of the complex, Chla611-Chla612. The results indicate that even small changes in the organization of the N-terminus, which can occur *in vivo* due to the reorganization of the membrane in stress conditions, could correspond to large changes in the Chla611-Chla612 coupling, making this site an excellent candidate for determining the poised state of LHCII^9^,^14^. On the other side, our results suggest that another putative quenching site involving Chl-Chl interactions, Chlb607-Chlb606, is unlikely to be involved in quenching. In addition, although our findings confirm that the observed Neo distortions correlate with different excitonic strengths at one possible quenching sites (Chla603-Lut 2), we did not find any evidence for the proposed direct cause-effect relationship^8^,^14^,^53^. The correlation of Neo with Chla603-Lut 2 and the large variations at the Chla611-Chla612-Lut 1 cluster also suggest that more than one quenching site can be present in LHCII. Our simulations clearly show that LHCII can exist in different conformational states characterized by large differences in the coupling strength between pigments, supporting the proposal that the NPQ “switch” can be driven by the conformational flexibility of LHC complexes.

## Methods

In the following we give an overview of the protocol employed to simulate our systems. Full details of the protocol and analyses are given in the Supplementary Information.

For all the MDs, we employed the GROMOS force-field (version 54a7^56^ for the apoprotein and 53a6^57^ for the pigments and lipids), which treats all the atoms explicitly except for some of the non-polar hydrogens^57^. See Supplementary Information for information on the development of the force-field parameters for LHCII cofactors. One monomer of LHCII from the crystal structure deposited by Liu *et al*. (Chain A, PDB 1RWT)^6^ was embedded in a lipid bilayer composed of POPC^58^ (344 total lipids), mimicking native membrane conditions^20^,^22^, and solvated in more than 15k SPC-water molecules at neutral physiologic salt concentration (10 mM Na^+^Cl^–^)^59^. We produced six independent simulations each lasting ~1 μs (simulations A, B and C and A, B, C-N-term) including all the crystallographic cofactors bound to LHCII (pigments, interstitial water molecules, DPPG). See Fig. 1. A for a scheme of the simulation box. Water molecules found in the X-ray structure^6^ were placed in the crystal although they were able to enter into the protein within 100 ns, as observed in an additional ~1 μs control simulation (MD No Water, Supplementary Figure S2.A-C and Video 1). Full set of simulations is described in detail in the Supplementary Information (Supplementary Table S1).

In all the simulations we first applied a careful multi-step equilibration (minimization, NVT and up to 140 ns NPT equilibration at 300 K) where position restraints (the position of selected atoms were restrained to the initial crystal coordinates) were gradually removed from the parts of the system we wanted to preserve from eventual distortions during the initial relaxation. These parts included chlorophyll rings, carotenoid chains, and the protein backbone (see full methods in SI). In three simulations (simulations A, B, C-N-term), just before the complete release of the position restraints, we removed constraints from the N-terminus, defined here as the first 39 residues (residues 14 to 53^6^). We then allowed the N-terminus to equilibrate for 100 ns before removing all other position restraints. This test was used to obtain a more complete sampling of this highly disordered domain^27^,^29^ and to test the effect of different conformations on the nearby chlorophylls. For all of the simulations, we then ran unbiased NPT simulations over timescales on the order of a microsecond. Parameters for the simulations and analyses protocols are given in the SI.

## Additional Information

**How to cite this article**: Liguori, N. *et al.* From light-harvesting to photoprotection: structural basis of the dynamic switch of the major antenna complex of plants (LHCII). *Sci. Rep.* **5**, 15661; doi: 10.1038/srep15661 (2015).

## Supplementary Material

Supplementary Information

Supplementary Video 1

Supplementary Video 2

Supplementary Video 3

Supplementary Video 4
