# Supplementary material for: From light-harvesting to photoprotection: structural basis of the dynamic switch of the major antenna complex of plants (LHCII)
Source: Sci Rep. 2015 Oct 23;5:15661. doi: 10.1038/srep15661 (PMC4616226; doi:10.1038/srep15661)
Supplement: Supplementary Information [file srep15661-s1.pdf]

From light-harvesting to photoprotection: structural basis of the dynamic switch of the major antenna complex of plants (LHCII)

Nicoletta Liguori[†], Xavier Periole[‡], Siewert-Jan Marrink[‡], Roberta Croce[†]*

[†]Department of Physics and Astronomy and Institute for Lasers, Life and Biophotonics, Faculty of Sciences, De Boelelaan 1081, 1081 HV, Amsterdam, The Netherlands & [‡]Groningen Biomolecular Sciences and Biotechnology Institute & Zernike Institute for Advanced Materials, University of Groningen, Nijenborgh 7, 9747 AG Groningen, The Netherlands

Supplementary Information (SI)

Materials and Methods.

In this work we performed classic Molecular Dynamics simulations (MDs) of a monomer of Light-Harvesting Complex II (LHCII) embedded in a lipid bilayer with explicit solvent. To model and simulate the system, and to analyze the large set of data, we made use of a set of pre-built packages and home-built tools listed below. We describe here the protocol followed to model, simulate and analyze the system. Additional analyses are also reported.

Software and tools. MDs were run on the GROMACS simulation package, version 4.6.3 (http://www.GROMACS.org/[1]). Simulations were visualized via the Visual Molecular Dynamics-VMD software (http://www.ks.uiuc.edu/Research/vmd/[2]). Analyses were run using the tools available on the GROMACS platform and through *ad hoc*-built codes.

LHCII pigment-protein monomeric complex modeling. All the simulations are based on the structure of a monomeric subunit of the Light-Harvesting Complex II (LHCII), crystallized by Liu and coworkers PDB 1RWT, chain A[3]. The monomer consists of 232 amino acids (residues 14 to 246), 8 chlorophyll a (Chla), 6 chlorophyll b (Chlb), 2 Luteins (Lut 1 and 2), 1 Violaxanthin (Vio), 1 Neoxanthin (Neo). Additionally present are 60 interstitial water molecules and one 1,2-dipalmitoyl-sn-glycero-3-phosphoglycerol (DPPG) lipid molecule[3].

The PDB coordinates of the protein were converted to a united-atom forcefield (GROMOS 54a7[4]) via the pdb2gmx tool of GROMACS[1]. All the titratable amino acids were considered to be in the standard protonation state at pH 7.

Of the total of 14 chlorophylls bound to LHCII, only a partial set of coordinates was available for the phytol tail of 4 of them (Chla 604, Chlb 605, Chlb 606 and Chla 614)[3]. The phytol tails are the most flexible part of the chlorophylls and in LHCII they occupy regions exposed to the environment, where mobility is expected to be high (see thermal factors in the main manuscript: Fig.2A-B, and below Fig.S1) making difficult to obtain the structure of the full phytol tail at high resolution[3,5,6]. We completed the missing coordinates of the partial Chla and Chlb by using the structure of one of the complete Chla or Chlb respectively from the same PDB file and copying the coordinates of the phytol tail, after overlapping the 4 tetrapyrrole-nitrogens of the complete Chl with those the partial Chl. B-factor is a strong indicator of conformational flexibility. Therefore, the high B-factor computed for the phytol tails in our MDs (and anticipated by their large B-factor in the crystal structure[3]), testify that over the long time scales investigated here (~1 μs) the choice of the initial coordinates of the phytol tails did not affect the sampling of their conformational space.

Pigments and DPPG force-field parametrization. Parameters for the full set of LHCII cofactors were derived compatibly with the GROMOS 53a6[7] forcefield, following the protocol we recently developed to build the topologies of the main cofactors of Photosystem II[8]. To summarize our methodology, after obtaining an initial topology using the Automated Topology Builder (ATB, atb.net[9]), the parameters obtained for the bonded terms of the potential (bonds, angles, proper and improper dihedrals) were refined based on GROMOS 53a6[7] forcefield building blocks. Partial charges were calculated via the GAMES-UK package[10], based on the Restricted Hartree-Fock method with 6-31G* basis set. Final charges were adjusted with respect to the symmetry of the molecule and to the united-atom GROMOS 53a6[7] model, for which the partial charges are summed and assigned to the carbon atom in all the CH_n groups (but not for aromatic or conjugated systems).

Similarly, we modeled Chlb based on the recently parametrized Chla[8]. The two molecules differ only at the C-7 position, where a formyl group in Chlb replaces the methyl group of Chla. Concerning the xanthophylls (Lut, Vio, Neo), most of the bond, angle, and dihedral values are compatible to the choices previously made for β-carotene[8]. Lut, Vio and Neo differ from β-carotene for the position of the double bond on the rings, the presence of hydroxyl groups (at both beta-rings), and that of epoxide groups at one (in Neo) or both beta-rings (in Vio). Additionally, Neo presents an allenic double bond.

The DPPG molecule was parametrized based on the dipalmitoyl-sn-glycero-3-phosphatidylcholine (DPPC) for the tails, and on the 1-palmitoyl-2-oleoyl-sn-glycero-3-phosphoglycerol (POPG) for the headgroup, developed by

Kukol et al.[11]. To model our homogeneous 1-palmitoyl-2-oleoyl-sn-glycero-3-phosphocholine (POPC) bilayer we used parameters from the same set of lipid topologies[11], which are available on http://lipidbook.bioch.ox.ac.uk.

LHCII-membrane system modeling. Embedding of the LHCII complex into a pre-equilibrated POPC bilayer was performed via a multi-step protocol. Relaxation of the bilayer prior to inserting the whole protein-cofactor complex allows a shorter equilibration time for the final system (LHCII + bilayer + explicit solvent). Therefore, our first step was to create a pre-equilibrated lipid bilayer satisfying the following features: the membrane should be wide enough to separate LHCII complex from its periodic image with at least ~6 lipids, and should present a "pore" large enough for the insertion of LHCII. To embed LHCII in the membrane we used genbox (tool of GROMACS[1]), which deletes any lipid or solvent molecules within van der Waals distance from the solute (in our case LHCII).

We first converted the LHCII-apoprotein structure (without cofactors) to a coarse grained force-field model (MARTINI[12]) via martinize.py tool (available at http://md.chem.rug.nl/cgmartini/). The MARTINI-LHCII apoprotein was later embedded into a MARTINI-POPC bilayer (two homogeneous layers of pure POPC in the ratio 204:204 per monolayer) with 5376 MARTINI-water beads via the insane.py tool[13] (available at http://md.chem.rug.nl/cgmartini/). After 500 steps of minimization (steepest descent), we then relaxed the system via 30 ns of NPT simulation (at a temperature of 323 K to equilibrate the bilayer faster). During all these steps, strong isotropic position restraints (10000 kJ mol^{-1} nm^{-2}) were applied to the whole protein to avoid shrinking of the protein structure (which in this simulation lacks all the cofactors). From the last snapshot of the MDs, we retrieved the coordinates of the bilayer alone (therefore presenting a pore in the membrane of the size of the CG-LHCII previously embedded). Finally, POPC bilayer was backmapped to the united atom resolution (GROMOS 53a6)[11] via the SUGARPIE tool[14].

Finally, after alignment of the GROMOS-LHCII with the pore in the GROMOS-POPC bilayer (via editconf tool, GROMACS[1]), we embedded the full LHCII in the membrane (via genbox tool, GROMACS[1]), and consequently solvated the system with water (via genbox) and ions (via genion tool, GROMACS[1]).

As described in the main manuscript, the final system consisted of one monomer of LHCII (including all the cofactors) embedded in a bilayer formed by 344 POPC molecules (a few lipids in van der Waals radius from LHCII were automatically deleted by genbox as previously explained), and solvated by more than 15k water molecules at neutral physiologic conditions (10 mM Na^+ Cl^-)[15].

Simulations. During minimization (steepest descent), NVT relaxation (10 ps), and the first part of the NPT equilibration period (40 ns), isotropic strong position restraints were applied to the protein and to its ligands. In this way, we aimed at relaxing the membrane and the solvent, minimizing perturbation of the protein and of the ligands crystal positions[3], as done by Ogata and coworkers for Photosystem II[16]. More in detail, position restraints were applied to the protein backbone, the chlorophyll-tetrapyrroles (but not the phytol tails), the carotenoid molecules and the DPPG lipid. Position restraints were set to a starting value of 10000 kJ mol^{-1} nm^{-2} and then gradually reduced to zero every 10 ns of NPT simulation (for a total of 40 ns simulated time), following the sequence 10000→ 1000→ 500→ 200→ 0 kJ mol^{-1} nm^{-2}. This equilibration protocol was repeated for the control simulation missing the initial crystallographic water molecules and the DPPG molecule (see main manuscript and Table S1). The final snapshot retrieved after the last 10 ns at 200 kJ mol^{-1} nm^{-2} (for the simulation started with the complete LHCII structure, including crystallographic water and DPPG), was used as starting conformation for the principal simulation A, B, and C (which have all been started from different random velocities). As anticipated in the main manuscript, from this conformation we started also the three control simulations (A-, B-, C- N-term) where the N-terminus (first 39 protein residues) was allowed to relax for additional 100 ns while the other cofactors and the protein backbone were kept constrained (force constant kept at 200 kJ mol^{-1} nm^{-2}), prior to removal of the remaining position restraints. Finally, a total of 7 independent NPT simulations were run up to ~1.1 μs (for the full set of MDs see Table S1). An integration time step of 2 fs was used for all the simulations, applying constraints on all the bonds (LINCS algorithm[17]). Particle Mesh Ewald (PME) scheme was used to treat long-range electrostatics, and cutoff values of 1 nm and 1.4 nm were selected respectively for short-range Coulomb and van der Waals interactions. Pressure was set to 1 bar under semi-isotropic coupling to a Parrinello-Rahman barostat[18], with relaxation time constant of 5 ps and compressibility of $4.5 \cdot 1o^{-5}$ bar^{-1}. Temperature was kept at 300 K via a Nose-Hoover thermostat[19]-scheme, with 0.5 ps time constant and with solvent, membrane and LHCII-complex coupled to the thermostat. All the simulations were run with Periodic Boundary Conditions (PBC).

In one of the control simulations (simulation No Water), the buried water molecules were absent and only the bulk water molecules were present in the starting simulation box. Using this control, we tested if the water is necessary for the stability of LHCII, since the water molecules are different in number and location in the two available LHCII crystal structures[3,20] (Figure S2.A). The absence of most of the water molecules in the latest crystal[20] might be due to limits in resolution of x-ray diffraction[21], and not to a functional reason. MDs represent a powerful tool to investigate the effective water sites[16,22]. Our simulation initially missing all the interstitial water molecules, converged to a re-hydrated LHCII in less than 100 ns (Video S1). In all the MDs, water molecules stably occupied the same sites (Figure S2.C), corresponding to the ones expected from the crystal of Liu et al[3], testifying that equilibrium is reached over the explored time-range. As hypothesized before[3], we find that most of the water molecules buried inside the protein are involved in chlorophyll coordination and stabilization of the helices (Figure S2.B).

Table S1. List of the simulations performed.

	MD No water	MDs A, B, C (Principal MDs)	MDs A, B, C-N-term
**Initial conditions**	-Only LHCII apoprotein and pigments are present, while crystallographic water molecules and DPPG molecule are absent. -Position restraints applied to all the protein backbone and pigments.	-All the crystallographic cofactors are present (pigments, interstitial water molecules, DPPG lipid). -Position restraints applied to all the protein backbone, pigments and DPPG.	-All the crystallographic cofactors are present (pigments, interstitial water molecules, DPPG lipid). -Position restraints applied to all the protein backbone and pigments. -Additional equilibration period after removal of position restraints from the N-terminus (first 39 residues).
**Total simulated time**	40 ns equilibration with position restraints + 1.08 µs unrestrained NPT.	40 ns equilibration with position restraints on LHCII and cofactors + starting from different random velocities, ~1 µs unrestrained NPT for each independent simulation (1.04 µs MD A, 1.05 µs MD B, 0.92 µs MD C).	40 ns equilibration with position restraints on LHCII and cofactors + 100 ns without position restraints on the N-terminus + starting from different random velocities, ~1 µs of unrestrained NPT for each independent simulation (0.95 µs A, 0.95 µs B, 0.91 µs C-N-term)
**Total simulations**	1	3	3

Analyses. Unless otherwise stated, the analyses were run over all the simulations except for the control simulation missing the crystallographic water molecules (MD No water, see Table S1). In most of the analyses, calculations have been performed on the whole trajectories after the first 400 ns of thermal equilibration. The description of the analyses is directly embedded in the caption of the respective figure.

B-factor (Figure 2 in the main manuscript, Figure S1 in the SI).
Root Mean Square Fluctuations (RMSF) of the atomic positions, were calculated via g_rmsf tool (GROMACS[1]) from the trajectories previously fitted (rotational + translational fit) onto the protein backbone and successively converted to B-factor for comparison with the crystal thermal factor. B-factor (B) is related to RMSF through the following formula: $B = \frac{8\pi^2}{3} RMSF^2$.

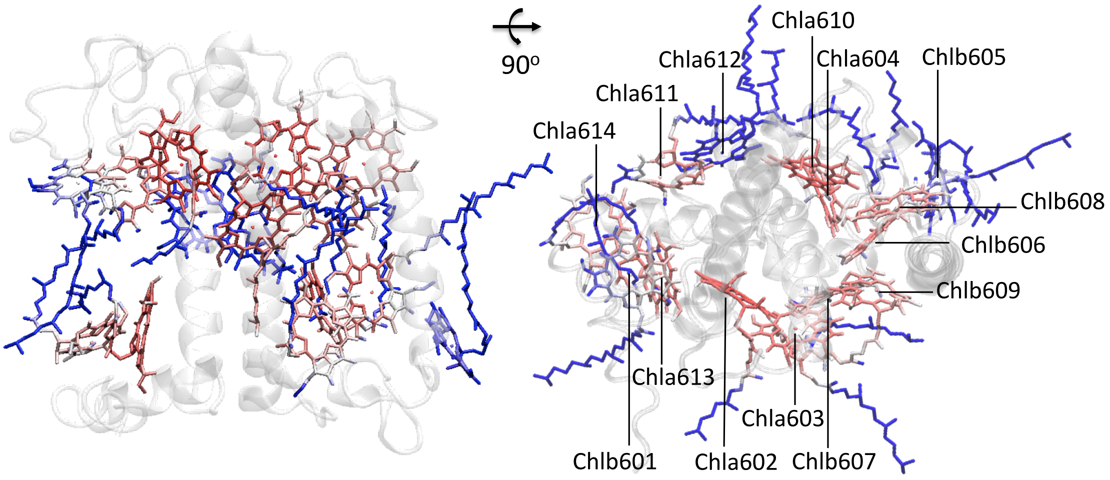

Figure S1. Chlorophyll organization in LHCII. Left: site view; Right: top view. LHCII apoprotein is shown in white. Chlorophylls are colored by their B-factor, with the most flexible parts are described in blue and the most rigid ones are described in red. B-factor range is here between 0 and 100 Å^2 as in Figure 2 in the main manuscript.

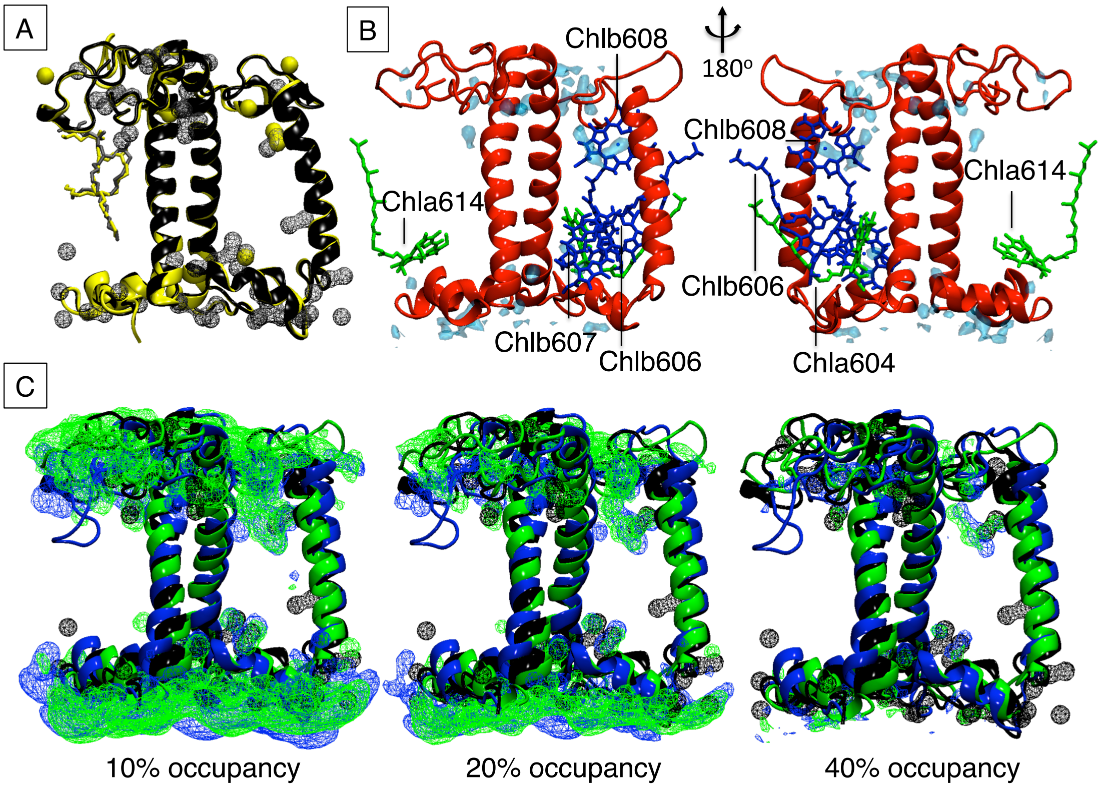

Figure S2.A-C. Water occupancy in LHCII. Here we calculated the water occupancy volume maps (at 1 Å resolution and combining all the frames by averaging) corresponding to the different populations of water molecules within 3 Å from LHCII protein, pigments and DPPG. Analysis of water occupancy in LHCII was performed via the Volmap tool (VMD)[2], over the full trajectory of the simulations after the first 400 ns.

In C the volume maps are represented as a mesh and correspond to the positions occupied by water molecules respectively for 10%, 20% and 40% of the total simulation time (after the first 400 ns). Three structures are shown: the Crystal structure (black), the simulation "No Water" (blue) and the principal simulation A (green).

In panel A the structural alignment of the protein (cartoon), water and DPPG molecules from the two available high resolution crystal structures of LHCII is shown: PDB-1RWT[3] in black with the water molecules described as a grid and PDB-2BHW[20] in yellow with the water molecules described by their van der Waals surfaces. In B the final protein structure from simulation A is shown, together with the water volume map corresponding to 40% occupancy in this simulation (the same as the green structure in C bottom right). The chlorophylls found within 3 Å from the reported water volumes are also shown. We found that DPPG helps to prevent large movements of the N-terminal domain which, in the absence of this lipid (simulation "No Water"), partially inserted in the membrane (as seen from the blue structure in C and in Video S1). The observed influence of DPPG on the N-terminus folding is particularly interesting considering that DPPG was shown to be necessary for LHC trimerization[23], which in turn is controlled by the N-terminus[24].

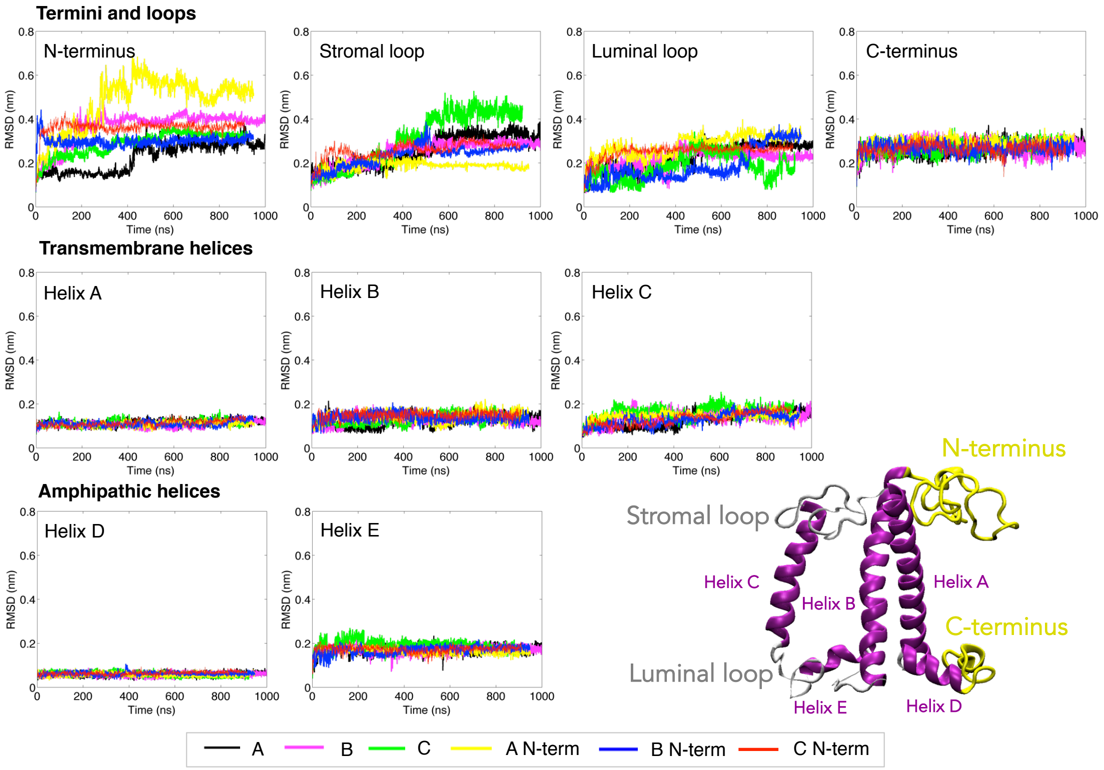

Figure S3. LHCII apoprotein dynamics as measured by the Root Mean Square Deviations (RMSD). RMSD for the single domains of LHCII protein (described by different colors in the protein structure on the bottom right side of the panel) were computed for each simulation against the common starting structure (reached at the end of the equilibration with position restraints over the whole LHCII complex with a force constant of 200 kJ mol^{-1} nm^{-2}, see above).

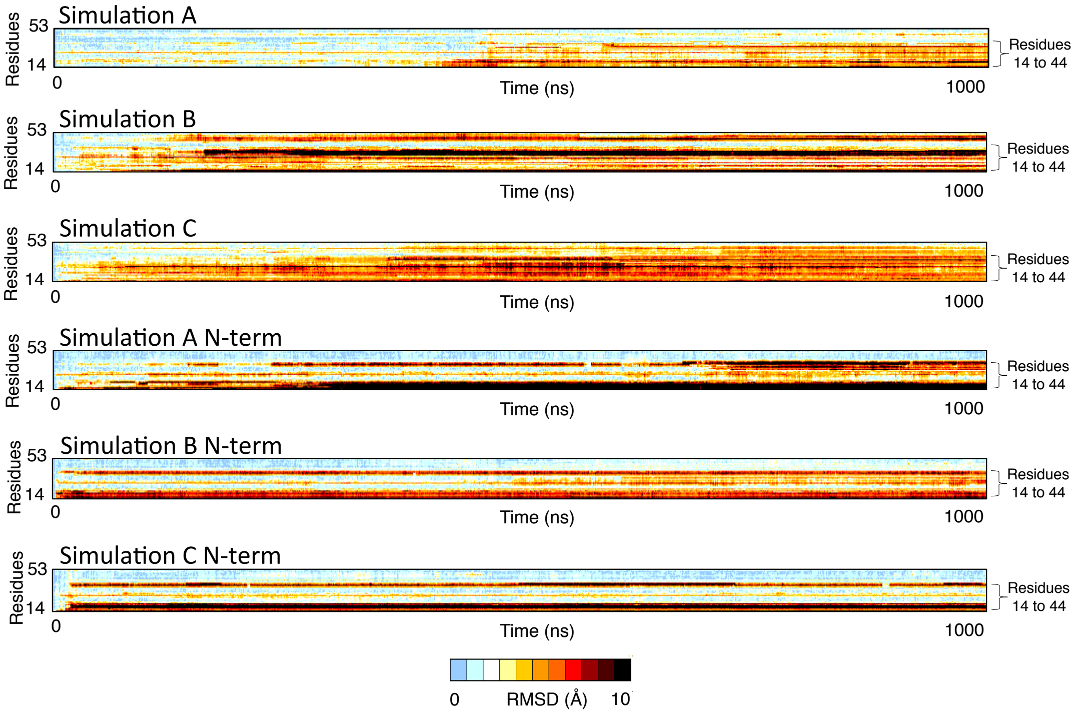

Figure S4. N-terminus conformational flexibility. Heat-Maps of the RMSD of the N-terminus calculated for each simulation against the common starting structure (similarly to what described above for Figure S3). The heat-maps show the time evolution of the RMSD per each single residue of this domain. Heat maps were constructed via the RMSD visualizer plugin of VMD[2].

Site N1 (Neo)	Lumen Side		Stroma side
Simulation	TYR112	Chla604	
A	19	14	-
B	80	31	-
C	58	16	-
A N-term	1	11	-
B N-term	40	12	-
C N-term	67	30	-
**Average**	**44**	**19**	-
Standard deviation	30	9	-
**Bonding Partners**	**TYR112: OH Neo: O1**	**Chla604: NA, NB, NC, ND, MG, O1A, O2A Neo: O1**	-

Site N1 (Neo)
Lumen side

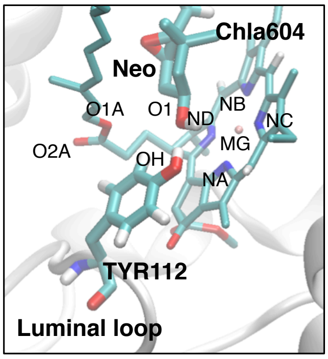

Table S2 and Figure S5. Neoxanthin H-bond to TYR112 and Chla604. H-bond occupancy is defined as the percentage of time that the H-bond is present between two partner atoms, over the total time of the simulation, excluding from the analysis the first 400 ns of simulation. The analyses were performed for each simulation. Via the HBonds plugin of VMD[2] a search was carried out over the atoms of the whole Neo molecule for binding partners amongst any LHCII residue and LHCII cofactor (pigments and DPPG) using the following criteria: Donor-Acceptor distance should be less than 3.5 Å (cut-off distance), Donor-H-Acceptor angle should be less than 30^0 (cut-off angle) and an occupancy higher than 10% should be present at least in four simulations. The is divided in two sections describing H-bond occupancy at the Lumen side and Stroma side of the membrane. The values of occupancy (% of the total simulated time after the first 400 ns), the average value (over the simulations) and the standard deviation associated to the average (%) are given per each simulation. At the bottom of each section the atoms involved in the H-bond with the hydroxyl group of each carotenoid, as determined by the analysis, are listed. In the Figure, the Neo carotenoid binding sites is shown**.** For each pair (carotenoid-residue) all the atoms possibly involved in the H-bond during the MD trajectory (see Table) are shown.

Carotenoid transition dipole moment (Figure 3 in the main manuscript, Table S3 in the SI). Carotenoid transition dipole moment (transition $S_2 \leftarrow S_0$) was considered oriented parallel to the central part of the polyene chain, as indicated by the green arrow in Figure 3 of the main manuscript and as modeled in previous studies[3,25,26]. We first employed a rotational + translational fit of the trajectories onto the protein backbone of the central helices A and B, for a scheme see Figure S2, taking as reference for the fit the same structure used for the RMSD analysis, see Figure S4. The z-axis of the box roughly coincides throughout the resulting trajectories with the 2-fold symmetry axis of the LHCII crystal (indicated in Figure 1.C, main manuscript). The angle between the dipole moment vector and the z-axis of the simulation box (often referred to as protein axis in the text), was calculated via dot product by using g_sgangle tool (GROMACS[1]). Time evolution of the angles respect to the protein axis is reported in Figure 3, of the main manuscript.

The final average angle ϑ_{MD} has been computed for each carotenoid and for each simulation (by averaging over the whole trajectory starting from 400 ns). The average variations of the angle respect to the crystal, $\Delta\vartheta$ (expressed in percentage), were calculated per each carotenoid as average over the ϑ_{MD} computed on the different simulations as:

$$\Delta\vartheta = \frac{(\vartheta_{crystal} - \vartheta_{MD})}{\vartheta_{crystal}} \cdot 100$$

where $\vartheta_{crystal}$ is the value of the angle in the crystal.
Additionally the final average angle, $\overline{\vartheta_{MD}}$, averaged over the final angles (ϑ_{MD}) of each simulation, and the standard deviation for this average were calculated. Also, we report the average variation $\Delta\bar{\vartheta}$ (expressed in percentage), respect to the crystal value which was calculated as:

$$\Delta\bar{\vartheta} = \frac{(\vartheta_{crystal} - \overline{\vartheta_{MD}})}{\vartheta_{crystal}} \cdot 100$$

Table S3. LHCII carotenoid angles respect to the protein axis. For each simulation we show the final average angle $\boldsymbol{\vartheta_{MD}}$ and the associated standard deviations (both expressed in degrees). In brackets the relative variations respect to the crystal value $\boldsymbol{\Delta\vartheta}$ (expressed in %) are reported. On the bottom of the Table we report per each carotenoid the average angle $\overline{\boldsymbol{\vartheta_{MD}}}$, calculated over the full set of average angles $\boldsymbol{\vartheta_{MD}}$, the associated standard deviation (both expressed in degrees) and the average variation from the crystal value $\boldsymbol{\Delta\overline{\vartheta}}$ (expressed in percentage) calculated as described above.

	Lut 1	Lut 2	Neo	Vio
Crystal (degrees)	74	69	61	21
A (degrees) (%)	72 ± 4 (-3)	66 ± 3 (-4)	77 ± 17 (26)	80 ± 18 (281)
B (degrees) (%)	67 ± 4 (-9)	69 ± 3 (0)	104 ± 8 (70)	96 ± 23 (357)
C (degrees) (%)	69 ± 4 (-7)	77 ± 4 (12)	96 ± 13 (57)	77 ± 17 (267)
A N-term (degrees) (%)	70 ± 4 (-5)	66 ± 2 (-4)	71 ± 13 (16)	88 ± 12 (319)
B N-term (degrees) (%)	70 ± 3 (-5)	72 ± 3 (4)	107 ± 5 (75)	46 ± 11 (119)
C N-term (degrees) (%)	72 ± 3 (-3)	66 ± 3 (-4)	77 ± 5 (26)	69 ± 25 (229)
Average angle (degrees)	70	69	89	76
Standard deviation (degrees)	2	4	16	17
Average variation (%)	-5	0	46	262

Excitonic coupling (Figure 4 in the main manuscript, Figure S6-7 and Table S4-5 in the SI).

Q_Y chlorophyll transition dipole moments were taken parallel to the nitrogen ND-NB axis, as in[3,25,27]. Carotenoid transition dipole moments were calculated as described above. Interaction energy (in cm^{-1}), also known as excitonic coupling strength, was calculated using the following formula:

$$V_{12} = \frac{f_1{}^2|\vec{\mu}_1||\vec{\mu}_2|}{\varepsilon_r} \cdot 5.04 \frac{(\hat{\mu}_1 \cdot \hat{\mu}_2 - 3 \cdot (\hat{\mu}_1 \cdot \hat{r}_{12})(\hat{\mu}_2 \cdot \hat{r}_{12}))}{R^3}$$

where f_1 is the local field correction factor, $|\vec{\mu}|$ the module of the transition dipole moment and $\hat{\mu}$ the normalized transition dipole moment vector, ε_r the relative dielectric constant (here equal to 2.4[27]), R the module of the distance between the center of the dipole moment vector and $\hat{r}_{12}$ the normalized distance vector (in nm), $(\hat{\mu}_1 \cdot \hat{\mu}_2 - 3 \cdot (\hat{\mu}_1 \cdot \hat{r}_{12})(\hat{\mu}_2 \cdot \hat{r}_{12}))$ is often referred to as orientation factor, k [28]. Dipole moment values were taken as 4, 3.4 and 4.5 Debye respectively for Chla, Chlb and Cars[3,25,27]. Angles between dipole vectors and distance vector (necessary for the dot products listed above) were calculated via g_sgangle tool (GROMACS[1]). Calculations of the final coupling strengths for the strongest coupled clusters, as in[3,25] (listed in Table S4-5 together with their standard deviation), were run via homebuilt codes over the full set of trajectories, and the final average value, E_{MD} (and the associated standard deviation) was calculated discarding the first 400 ns of each simulation. Per each E_{MD} obtained in each simulation and per each Chl-Chl pair, we then calculated the variations of the coupling strength respect to the crystal (ΔE), which are expressed in percentages, similarly to what explained above for $\Delta\vartheta$. Also, the final average variation, $\Delta\bar{E}$, was calculated for each Chl-Chl pair similarly to $\Delta\bar{\vartheta}$, based on the final average coupling value $\bar{E}$ (average over the various E_{MD}).

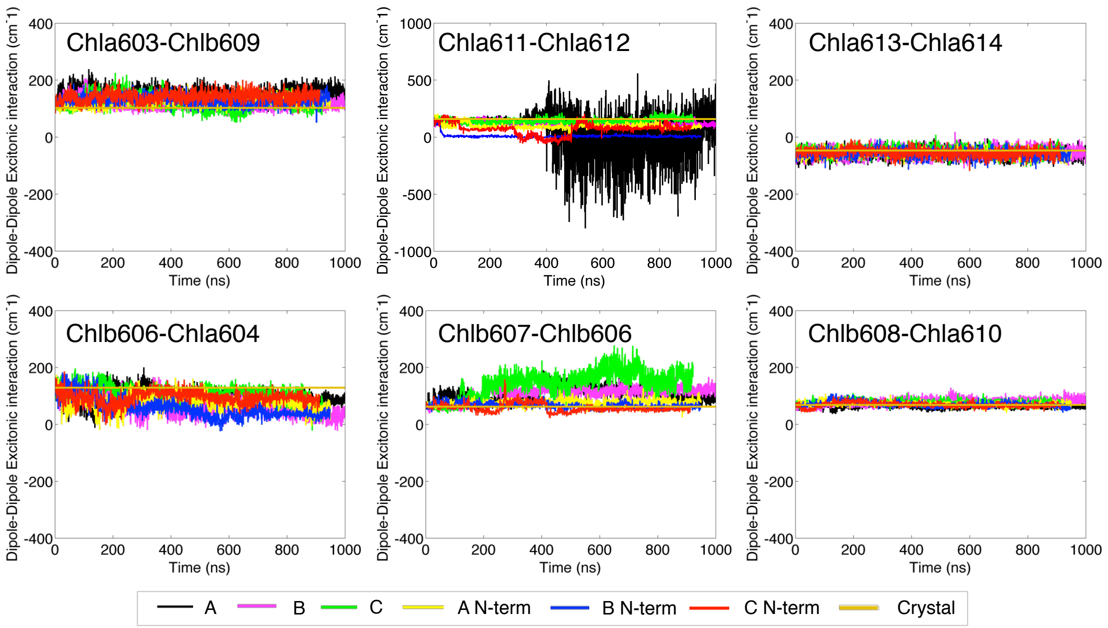

Figure S6. Time evolution of Chl-Chl interaction energy in the different simulations. Full set of time-evolution excitonic coupling strengths calculated as described above. The corresponding crystal values are shown as a straight line in gold, as indicated in the legend.

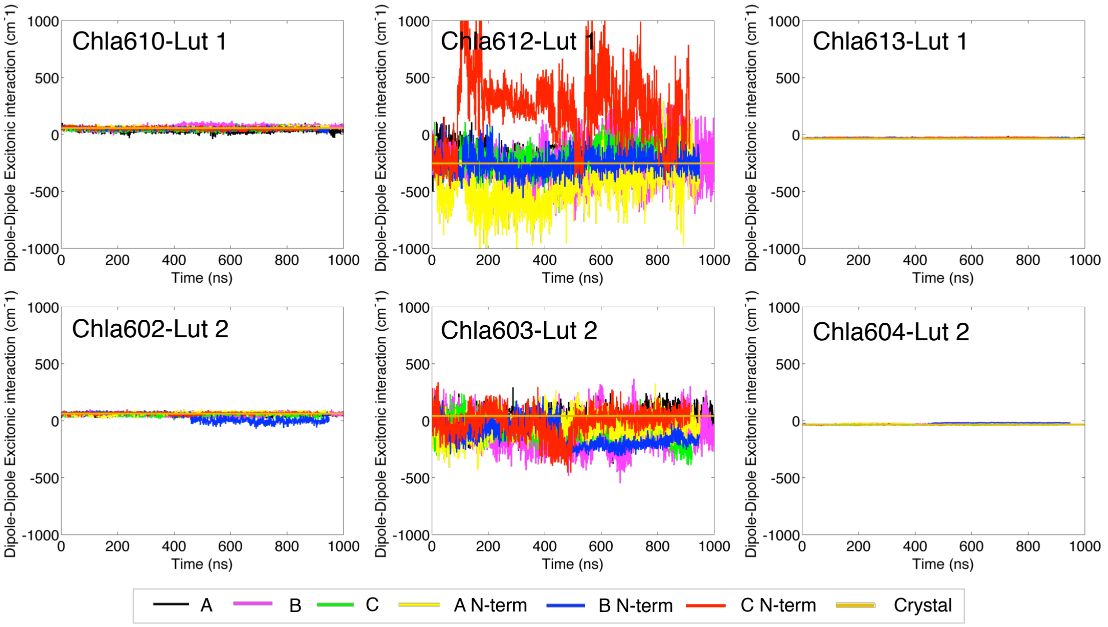

Figure S7. Time evolution of Chl-Car interaction energy in the different simulations. Full set of time-evolution excitonic coupling strengths calculated as described above. The corresponding crystal values are shown as a straight line in gold, as indicated in the legend.

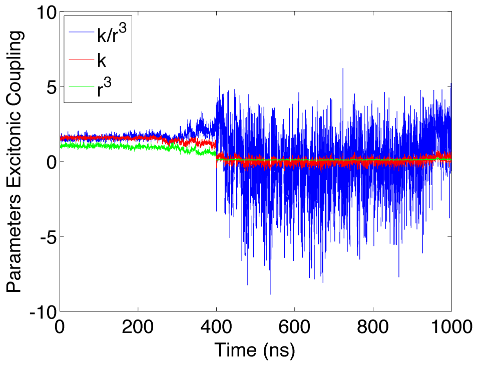

Figure S8. Time evolution of Chla611-Chla612 orientation and distance in simulation A. Excitonic coupling in the point-dipole approximation (here used to calculate all the coupling values, see Methods) is proportional to the orientation factor k and inversely proportional to the cube of the distance between the dipole moments (r^3). In the figure these values are plotted separately. It can be seen that the large variations observed in this simulation are mostly due to changes in orientation

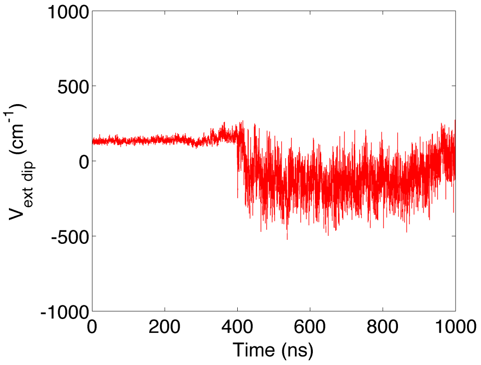

Figure S9. Excitonic coupling computed via the extended dipole method for the Chla611-Chla612 cluster in simulation A. Excitonic coupling was calculated as $V_{ext\ dip} = \frac{1}{4\pi\varepsilon_0}\sum_{ij=\pm}\frac{q_i^1 q_j^2}{|r_i^1 - r_j^2|}$ [29,30] where the partial charge q is equal to $q = \frac{\mu}{d}$ (with μ being the dipole taken equal to 4 Debye as in the point-dipole approximation and d the distance between the NB-ND nitrogens of the same chlorophyll) and $|r_i^1 - r_j^2|$ is the distance between the partial charges located in this approximation on the NB and ND nitrogens of each chlorophyll. ε_0 is the vacuum permittivity. It should be noted that, in this approximation, partial charges have been placed on the nitrogens, which might result in an underestimation of the actual dipole extent. As reported in the main manuscript, the fluctuations in coupling are reduced compared to the point-dipole method (see Figure 4 and Supplementary Figure S6). However, the trend over time computed via the point-dipole method is the same, confirming that the rearrangement of this chlorophyll pair leads to a strong reduction of their interaction energy.

Table S4. Chl-Chl excitonic coupling strengths. In the Table we report the results of the analysis described above. For each Chl-Chl pair and for each simulation, we list the average excitonic coupling strength, $\mathbf{E_{MD}}$, the associated standard deviation (both expressed in cm^{-1}) and in brackets the variation in the coupling strength ($\boldsymbol{\Delta E}$) respect to the crystal, expressed in percentage. On the bottom part of the Table, we report the final average coupling $\bar{\mathbf{E}}$, calculated over the whole set of $\mathbf{E_{MD}}$, and the associated standard deviation (both expressed in cm^{-1}). Finally, we report the average variation in coupling strength $\boldsymbol{\Delta \bar{E}}$ (expressed in percentage).

	Chla603-Chlb609	Chla611-Chla612	Chla613-Chla614	Chlb606-Chla604	Chlb607-Chlb606	Chlb608-Chla610
Crystal	103	158	-47	129	62	68
A (cm^{-1}) (%)	147 ± 20 (43)	34 ± 187 (-103)	-57 ± 15 (21)	100 ± 18 (-22)	109 ± 26 (76)	63 ± 6 (-7)
B (cm^{-1}) (%)	110 ± 13 (7)	110 ± 17 (-34)	-52 ± 15 (11)	59 ± 27 (-54)	119 ± 13 (92)	82 ± 10 (21)
C (cm^{-1}) (%)	121 ± 23 (17)	143 ± 21(-8)	-51 ± 15 (9)	101 ± 23 (-22)	160 ± 30 (158)	76 ± 8 (12)
A N-term (cm^{-1}) (%)	129 ± 14 (25)	92 ± 15 (-41)	-58 ± 13 (23)	75 ± 23 (-42)	78 ± 12 (26)	71 ± 7 (4)
B N-term (cm^{-1}) (%)	130 ± 18 (26)	7 ± 8 (-95)	-58 ± 15 (23)	45 ± 22 (-65)	65 ± 9 (5)	69 ± 8 (1)
C N-term (cm^{-1}) (%)	143 ± 18 (39)	59 ± 48 (-56)	-55 ± 15 (17)	96 ± 20 (-26)	57 ± 15 (-8)	69 ± 8 (1)
Average coupling (cm^{-1})	130	74	-55	79	98	72
Standard deviation (cm^{-1})	14	50	3	24	39	7
Average variation (%)	26	-53	17	-39	58	6

Table S5. Chl-Car excitonic coupling strengths. The results of the analysis on each Chl-Car strong excitonic couples are reported similarly to what described for Table S4.

	Chla610-Lut 1	Chla612-Lut 1	Chla613-Lut 1	Chla602-Lut 2	Chla603-Lut 2	Chla604-Lut 2
Crystal	53	-240	-34	59	40	-32
A (cm^{-1}) (%)	39 ± 18 (-26)	-143 ± 70 (-40)	-30 ± 3 (-12)	56 ± 11 (-5)	-15 ± 86 (-138)	-35 ± 2 (9)
B (cm^{-1}) (%)	69 ± 16 (30)	-245 ± 159 (2)	-31 ± 3 (-9)	56 ± 16 (-5)	-119 ± 121 (-398)	-31 ± 3 (-3)
C (cm^{-1}) (%)	45 ± 13 (-15)	-184 ± 105 (-23)	-31 ± 3 (-9)	57 ± 12 (-3)	-86 ± 75 (-315)	-36 ± 3 (13)
A N-term (cm^{-1}) (%)	62 ± 9 (17)	-452 ± 181 (88)	-32 ± 3 (-6)	71 ± 10 (20)	-27 ± 87 (-168)	-27 ± 4 (-16)
B N-term (cm^{-1}) (%)	52 ± 12 (-2)	-255 ± 84 (6)	-28 ± 4 (-18)	22 ± 36 (-63)	-135 ± 108 (-438)	-23 ± 7 (-28)
C N-term (cm^{-1}) (%)	52 ± 12 (-2)	223 ± 239 (-193)	-27 ± 3 (-21)	65 ± 10 (10)	-15 ± 118 (-138)	-35 ± 3 (9)
Average coupling (cm^{-1})	53	-176	-30	55	-66	-31
Standard deviation (cm^{-1})	11	223	2	17	54	5
Average variation (%)	0	-27	-12	-7	-265	-3

N-terminus to DPPG distance and Chlb607-formyl to GLN131 oxygen distance (Figure S8 and Table S6). The distance between the most flexible part of the N-terminus (residues 14-44, see Figure S4) and the DPPG headgroup, and the distance between the Chlb607-formyl hydrogen and the GLN131 oxygen were calculated using the g_dist tool of GROMACS[1]. The associated final average values ($d_{\text{N-terminus-DPPG}}$, $d_{\text{Chlb607-GLN131}}$) and the associated standard deviations (calculated starting from 400 ns of trajectory), the relative variations per each simulation $\Delta d_{\text{N-terminus-DPPG}}$ and $\Delta d_{\text{Chlb607-GLN131}}$ and the final average variations $\Delta \bar{d}_{N-terminus-DPPG}$ and $\Delta \bar{d}_{Chlb607-GLN131}$ were calculated as described above for the carotenoid dipole moment analysis. The results are summarized in Table S6.

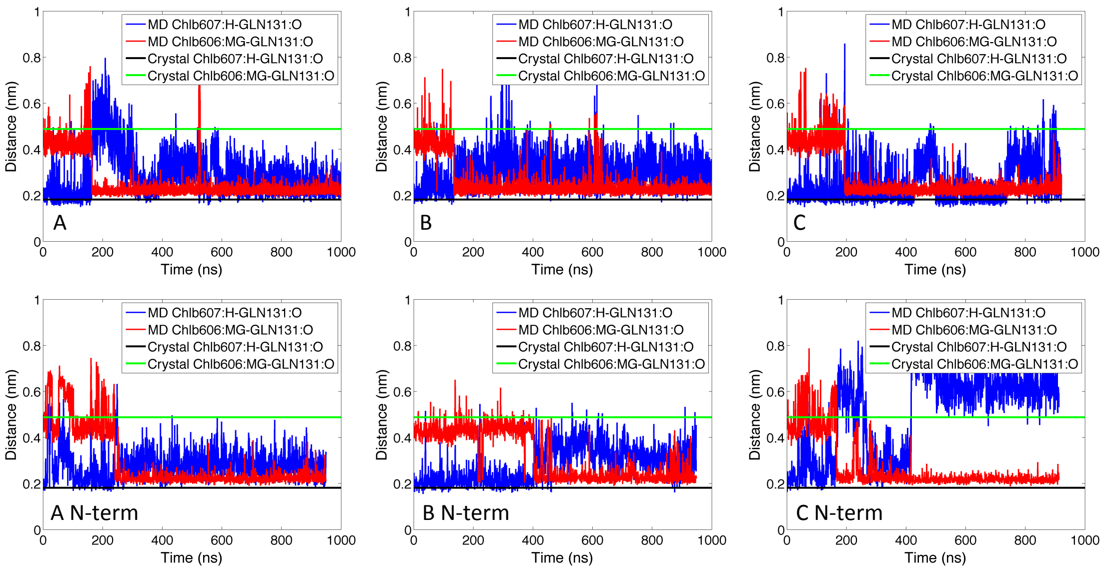

Figure S10. H-bond at the Chlb607 site. We here report the results of the analysis of distances between the Chlb607-formyl hydrogen and the GLN131 oxygen (Chlb607:H-GLN131:O) and between the Chlb606-central magnesium and the GLN131 oxygen (Chlb606:MG-GLN131:O). The Chlb606:MG-GLN131:O distance was calculated as described above for the Chlb607:H-GLN131:O group. Video S3 shows an example extracted from simulation A (here shown in the upper left side of the panel) of the event of H-bond loss between the Chlb607-formyl group and the GLN131 oxygen. GLN131 oxygen, after an initial period of equilibration, switches to coordinate the central magnesium of Chlb606. In the plots the sudden decrease of distance between the Chlb606-magnesium and the GLN131 oxygen, and the simultaneous increase of distance between the Chlb607-formyl group and the GLN131 oxygen (H-bond loss) are clearly visible. Chlb607:H-GLN131:O distance varies in average 94% from the initial distance value (see Table S5).

Table S6. Analysis of the N-terminus to DPPG distance and Chlb607-formyl to GLN131 oxygen distance. In the table we report per each simulation, the average values and associated standard deviations (expressed in nm) and the variations respect to the crystal value (expressed in percentage) for the N-terminus to DPPG and Chlb607-formyl to GLN131 oxygen distances ($d_{\text{N-terminus-DPPG}}$, $d_{\text{Chlb607-GLN131}}$, $\Delta d_{\text{N-terminus-DPPG}}$ and $\Delta d_{\text{Chlb607-GLN131}}$). At the bottom of the Table, the final average distance and the associated standard deviations (expressed in nm) and the final average variation respect to the crystal, $\bar{d}_{\text{N-terminus-DPPG}}$, $\bar{d}_{Chlb607-GLN131}$, $\Delta\bar{d}_{\text{N-terminus-DPPG}}$ and $\Delta\bar{d}_{Chlb607-GLN131}$, calculated similarly to what described for the carotenoid transition dipole moment (see above) are reported.

	$\Delta d_{\text{N-terminus-DPPG}}$	$\Delta d_{\text{Chlb607-GLN131}}$
**Crystal**	1.29	0.18
**A (nm) (%)**	0.71 ± 0.06 (-45)	0.28 ± 0.06 (52)
**B (nm) (%)**	1.11 ± 0.07 (-14)	0.30 ± 0.06 (67)
**C (nm) (%)**	1.21 ± 0.11 (-6)	0.27 ± 0.09 (47)
**A N-term (nm) (%)**	1.12 ± 0.09 (-13)	0.28 ± 0.05 (55)
**B N-term (nm) (%)**	0.71 ± 0.03 (-45)	0.33 ± 0.06 (79)
**C N-term (nm) (%)**	0.99 ± 0.15 (-23)	0.63 ± 0.10 (246)
**Average distance (nm)**	0.98	0.35
**Standard deviation (nm)**	0.22	0.14
**Average variation (%)**	-24	94

	Chla603-Chlb609	Chla611-Chla612	Chla613-Chla614	Chlb606-Chla604	Chlb607-Chlb606	Chlb608-Chla610
$\Delta d_{N\text{-terminus-DPPG}}$	-0.74	0.99	-0.77	0.48	0.19	0.50
$\Delta\vartheta_{neo}$	-0.04	-0.25	0.22	-0.77	0.43	0.57
$\Delta d_{Chlb607\text{-}GLN131}$	0.58	-0.29	0.34	-0.15	-0.34	-0.06

	Chla610-Lut 1	Chla612-Lut 1	Chla613-Lut 1	Chla602-Lut 2	Chla603-Lut 2	Chla604-Lut 2
$\Delta d_{N\text{-terminus-DPPG}}$	0.40	0.18	0.69	0.59	0.32	0.29
$\Delta\vartheta_{neo}$	0.18	0.06	-0.45	-0.66	-0.98	-0.30
$\Delta d_{Chlb607\text{-}GLN131}$	0.01	-0.82	-0.82	0.12	-0.03	0.19

Table S7. Correlation between conformational changes and excitonic coupling. Here the correlation coefficients (Pearson) between a set of conformational changes and the variations of the excitonic couplings (ΔE) are reported. With this analysis we tested the hypothesis that a correlation exists between the ensemble of possible coupling states obtained from the relaxed structures of LHCII in the membrane (in the different simulations), and the various conformations reached after the systematic conformational changes reported.

The conformational changes that we considered are the decrease of distance between the N-terminus and the DPPG headgroup, $\Delta d_{N\text{-terminus-DPPG}}$, the Neo distorsion calculated as variation in the dipole moment angle respect to the z-axis of the protein, $\Delta\vartheta_{neo}$, and the increase of distance between the Chlb607-formyl hydrogen and the GLN131 oxygen (H-bond loss), $\Delta d_{Chlb607\text{-}GLN131}$.

Per each single Chl-Chl or Chl-Car exciton dimer, we calculated the Pearson coefficient over the ensemble of different ΔE and $\Delta d_{N\text{-terminus-DPPG}}$, $\Delta\vartheta_{neo}$ or $\Delta d_{Chlb607\text{-}GLN131}$ reported from the simulations A, B, C and A-, B-, C- N-term. The values used in this analysis are reported in Table S3-7. As reported in the main manuscript (Figure 5), the (0,0) point per each serie of data represents the crystal state.

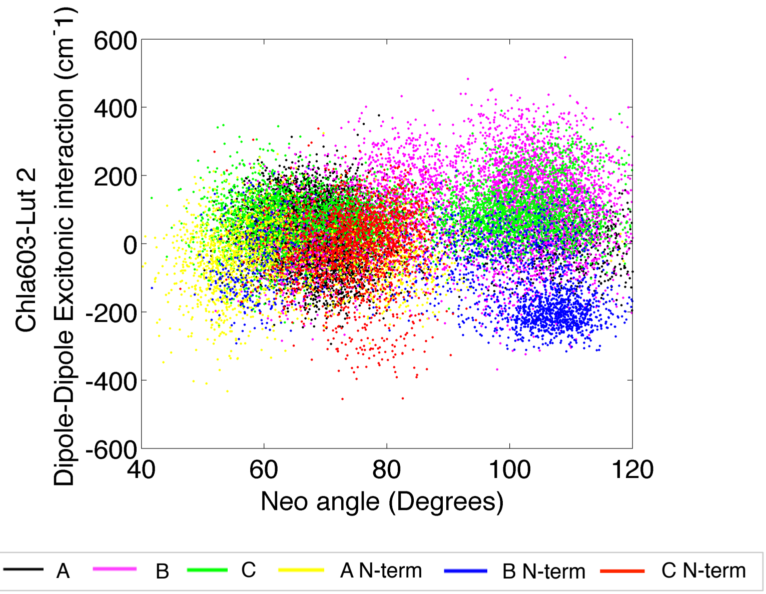

	A	B	C	A N-term	B N-term	C N-term
**Correlation**	-0.16	0,22	0.26	0,05	-0,33	0.04

Figure S11 and Table S8. Correlation between the Neo bending motion and the putative quenching site Chla603-Lut 2. For each single simulation the correlation over the full trajectory between the Neo tilt angle (reported in Figure 3) and the Chla603-Lut 2 time evolutions (reported in Figure 4 and Figure S7) is shown. For each simulation we calculated the Pearson coefficients, which are then summarized in Table S8.

Videos. All the videos have been rendered via VMD[2] software.

Video V1. LHCII re-hydration. In this video, the full trajectory of the MD "No Water" is shown (for a total of ~1.1 µs, see Table S1). This simulation of the LHCII complex has been started in absence of the crystallographic waters (which are then present in the bulk water for a total of ~15K water molecules) and of the lipid DPPG. In the video the structural alignment of the MD-protein (red) and LHCII from the crystal[3] (1RWT PDB, chain A, black) is shown. The water molecules are shown as van der Waals-glossy beads (MD No water) and as black wireframe (crystal). The water molecules within 3 Å from the protein are shown. The re-hydration of LHCII apoprotein takes place in less than 100 ns in this simulation.

Video V2. Violaxanthin detachment and Neoxanthin distortion. In this video we show the full trajectories, after fitting them onto the C_α carbon atoms of the proteins, of the three principal simulations (A, B and C). The protein structure is represented in black for simulation A, magenta for simulation B, green for simulation C. On the left side of the protein structure the Violaxanthin molecule (left) and the DPPG lipid (right) for each simulation are shown. On the right side of the protein the carotenoid Neoxanthin is shown. The color code for the cofactors is the same as for the protein. In the Video two main events are shown: the detachment of Violaxanthin (Figure 3 and Table S3) and distortion of Neoxanthin (Figure 3 and Table S3).

Video V3. H-bond loss at the Chlb607-Chlb606 site. In the Video, extracted from the full trajectory of simulation A, loss of the H-bond between Chlb607-formyl group and the GLN131 oxygen is shown (see Figure S8). Also, it is possible to see the switch of the GLN131 oxygen that during the simulation becomes the ligand for the central magnesium of Chlb606 (see Figure S8). Chlb607 and Chlb606 are respectively in purple and yellow. GLN131 and the formyl group of the two Chls are colored based on their atoms (red for oxygen, white for hydrogen, cyan for carbon and blue for nitrogen). The protein in the background is rendered in transparent green. Water molecules within 3 Å from the Chl607-Chl606 group and from GLN131 are also shown.

Video V4. Energy disorder at the Chl611-Chl612 site. In the Video we report the event registered at the Chla611-Chla612 site showing the full trajectory of simulation A. In this simulation a strong deviation from the crystal value of the excitonic coupling strength for this cluster of Chls is observed (see Figure 4 and S6, and Table S4). The Video shows that this disorder is originated by a variation of the relative distance and orientation of the two Chls. It is shown that DPPG interacts with Chl611 and with various residues at the N-terminus of the protein. Chla612 is in magenta and Chla611 is in gold. DPPG lipid is colored based on each constituent atom (see Video V3 caption). In the background the LHCII apoprotein is in transparent green. All the residues of the N-terminus within 3 Å from the DPPG headgroup are shown. It is then possible to see that going toward the end of the simulation (1.04 µs, see Table S1) the network of interactions of the DPPG with the N-terminus largely increases.

References.

1. Van Der Spoel, D. *et al.* GROMACS: fast, flexible, and free. *J. Comput. Chem.* **26,** 1701–18 (2005).
2. Humphrey, W., Dalke, A. & Schulten, K. VMD: visual molecular dynamics. *J. Mol. Graph.* **14,** 33–38 (1996).
3. Liu, Z. *et al.* Crystal structure of spinach major light-harvesting complex at 2.72 A resolution. *Nature* **428,** 287–92 (2004).
4. Schmid, N. *et al.* Definition and testing of the GROMOS force-field versions 54A7 and 54B7. *Eur. Biophys. J.* **40,** 843–856 (2011).
5. Barros, T., Royant, A., Standfuss, J., Dreuw, A. & Kühlbrandt, W. Crystal structure of plant light-harvesting complex shows the active, energy-transmitting state. *EMBO J.* **28,** 298–306 (2009).
6. Pan, X. *et al.* Structural insights into energy regulation of light-harvesting complex CP29 from spinach. *Nat. Struct. Mol. Biol.* **18,** 309–15 (2011).
7. Oostenbrink, C., Soares, T. A., van der Vegt, N. F. A. & van Gunsteren, W. F. Validation of the 53A6 GROMOS force field. *Eur. Biophys. J.* **34,** 273–84 (2005).
8. De Jong, D. H. *et al.* Atomistic and Coarse Grain Topologies for the Cofactors Associated with the Photosystem II Core Complex. *J. Phys. Chem. B* **119,** 7791–7803 (2015).
9. Malde, A. K. *et al.* An Automated Force Field Topology Builder (ATB) and Repository: Version 1.0. *J. Chem. Theory Comput.* **7,** 4026–4037 (2011).
10. Guest *, M. F. *et al.* The GAMESS-UK electronic structure package: algorithms, developments and applications. *Mol. Phys.* **103,** 719–747 (2005).
11. Kukol, A. Lipid Models for United-Atom Molecular Dynamics Simulations of Proteins. *J. Chem. Theory Comput.* **5,** 615–626 (2009).
12. Marrink, S. J. & Tieleman, D. P. Perspective on the Martini model. *Chem. Soc. Rev.* **42,** 6801–22 (2013).
13. Wassenaar, T. A., Ingólfsson, H. I., Böckmann, R. A., Tieleman, D. P. & Marrink, S. J. Computational lipidomics with insane: a versatile tool for generating custom membranes for molecular simulations. *J. Chem. Theory Comput.* 150410125128004 (2015). doi:10.1021/acs.jctc.5b00209

14. Brocos, P., Mendoza-Espinosa, P., Castillo, R., Mas-Oliva, J. & Piñeiro, Á. Multiscale molecular dynamics simulations of micelles: coarse-grain for self-assembly and atomic resolution for finer details. *Soft Matter* **8,** 9005 (2012).
15. Gomez, J. M., Jimenez, A., Olmos, E. & Sevilla, F. Location and effects of long-term NaCl stress on superoxide dismutase and ascorbate peroxidase isoenzymes of pea (Pisum sativum cv. Puget) chloroplasts. *J. Exp. Bot.* **55,** 119–30 (2004).
16. Ogata, K., Yuki, T., Hatakeyama, M., Uchida, W. & Nakamura, S. All-atom molecular dynamics simulation of photosystem II embedded in thylakoid membrane. *J. Am. Chem. Soc.* **135,** 15670–3 (2013).
17. Hess, B., Bekker, H., Berendsen, H. J. C. & Fraaije, J. G. E. M. LINCS: a linear constraint solver for molecular simulations. *J. Comput. Chem.* **18,** 1463–1472 (1997).
18. Parrinello, M. Polymorphic transitions in single crystals: A new molecular dynamics method. *J. Appl. Phys.* **52,** 7182 (1981).
19. Nosé, S. A unified formulation of the constant temperature molecular dynamics methods. *J. Chem. Phys.* **81,** 511 (1984).
20. Standfuss, J., Terwisscha van Scheltinga, A. C., Lamborghini, M. & Kühlbrandt, W. Mechanisms of photoprotection and nonphotochemical quenching in pea light-harvesting complex at 2.5 A resolution. *EMBO J.* **24,** 919–28 (2005).
21. Yu, B., Blaber, M., Gronenborn, A. M., Clore, G. M. & Caspar, D. L. D. Disordered water within a hydrophobic protein cavity visualized by x-ray crystallography. *Proc. Natl. Acad. Sci.* **96,** 103–108 (1999).
22. Miao, Y. & Baudry, J. Active-site hydration and water diffusion in cytochrome P450cam: a highly dynamic process. *Biophys. J.* **101,** 1493–503 (2011).
23. Hobe, S., Prytulla, S., Kühlbrandt, W. & Paulsen, H. Trimerization and crystallization of reconstituted light-harvesting chlorophyll a/b complex. *EMBO J.* **13,** 3423–9 (1994).
24. Hobe, S., Foerster, R., Klingler, J. & Paulsen, H. N-Proximal Sequence Motif in Light-Harvesting Chlorophyll a/b-Binding Protein Is Essential for the Trimerization of Light-Harvesting Chlorophyll a/b Complex. *Biochemistry* **34,** 10224–10228 (1995).
25. Georgakopoulou, S. *et al.* Understanding the changes in the circular dichroism of light harvesting complex II upon varying its pigment composition and organization. *Biochemistry* **46,** 4745–54 (2007).
26. Georgakopoulou, S., van Grondelle, R. & van der Zwan, G. Circular dichroism of carotenoids in bacterial light-harvesting complexes: experiments and modeling. *Biophys. J.* **87,** 3010–22 (2004).
27. Van Amerongen, H. & van Grondelle, R. Understanding the Energy Transfer Function of LHCII, the Major Light-Harvesting Complex of Green Plants †. *J. Phys. Chem. B* **105,** 604–617 (2001).
28. Van Amerongen, H., Valkunas, L. & Van Grondelle, R. *Photosynthetic excitons*. (World Scientific, 2000).
29. Madjet, M. E., Abdurahman, A. & Renger, T. Intermolecular coulomb couplings from ab initio electrostatic potentials: application to optical transitions of strongly coupled pigments in photosynthetic antennae and reaction centers. *J. Phys. Chem. B* **110,** 17268–81 (2006).
30. Müh, F., Madjet, M. E.-A. & Renger, T. Structure-based identification of energy sinks in plant light-harvesting complex II. *J. Phys. Chem. B* **114,** 13517–35 (2010).
